# Prediction of benign and malignant ground glass pulmonary nodules based on multi-feature fusion of attention mechanism

**DOI:** 10.3389/fonc.2024.1447132

**Published:** 2024-10-09

**Authors:** Heng Deng, Wenjun Huang, Xiuxiu Zhou, Taohu Zhou, Li Fan, Shiyuan Liu

**Affiliations:** ^1^ School of Medicine, Shanghai University, Shanghai, China; ^2^ Department of Radiology, The Second People’s Hospital of Deyang, Deyang, Sichuan, China; ^3^ Department of Radiology, Second Affiliated Hospital of Naval Medical University, Shanghai, China

**Keywords:** ground-glass nodule, deep learning, computed tomography (CT), attention mechanism, feature fusion

## Abstract

**Objectives:**

The purpose of this study was to develop and validate a new feature fusion algorithm to improve the classification performance of benign and malignant ground-glass nodules (GGNs) based on deep learning.

**Methods:**

We retrospectively collected 385 cases of GGNs confirmed by surgical pathology from three hospitals. We utilized 239 GGNs from Hospital 1 as the training and internal validation set, and 115 and 31 GGNs from Hospital 2 and Hospital 3, respectively, as external test sets 1 and 2. Among these GGNs, 172 were benign and 203 were malignant. First, we evaluated clinical and morphological features of GGNs at baseline chest CT and simultaneously extracted whole-lung radiomics features. Then, deep convolutional neural networks (CNNs) and backpropagation neural networks (BPNNs) were applied to extract deep features from whole-lung CT images, clinical, morphological features, and whole-lung radiomics features separately. Finally, we integrated these four types of deep features using an attention mechanism. Multiple metrics were employed to evaluate the predictive performance of the model.

**Results:**

The deep learning model integrating clinical, morphological, radiomics and whole lung CT image features with attention mechanism (CMRI-AM) achieved the best performance, with area under the curve (AUC) values of 0.941 (95% CI: 0.898-0.972), 0.861 (95% CI: 0.823-0.882), and 0.906 (95% CI: 0.878-0.932) on the internal validation set, external test set 1, and external test set 2, respectively. The AUC differences between the CMRI-AM model and other feature combination models were statistically significant in all three groups (all p<0.05).

**Conclusion:**

Our experimental results demonstrated that (1) applying attention mechanism to fuse whole-lung CT images, radiomics features, clinical, and morphological features is feasible, (2) clinical, morphological, and radiomics features provide supplementary information for the classification of benign and malignant GGNs based on CT images, and (3) utilizing baseline whole-lung CT features to predict the benign and malignant of GGNs is an effective method. Therefore, optimizing the fusion of baseline whole-lung CT features can effectively improve the classification performance of GGNs.

## 1 Introduction

Lung cancer remains the deadliest cancer worldwide and early detection is crucial for its treatment (1, 2). In the early stages of lung cancer, pulmonary lesions often manifest as pulmonary nodules (3), among which ground-glass nodules (GGNs) are one of the main manifestations (4, 5). For patients with early malignant lung diseases accompanied by ground-glass nodules, intervention therapy can achieve a cure rate of over 80% (6). With the widespread application of high-resolution CT and low-dose CT (LDCT) for lung cancer screening, the detection rate of pulmonary ground-glass nodules (GGNs) has significantly increased (7, 8). However, due to the large number of GGNs confirmed as benign by histopathology, the imaging features between early-stage lung adenocarcinoma and benign GGNs are very similar (9). Therefore, distinguishing between benign and malignant GGNs is challenging (10, 11).

In recent years, numerous researchers have developed various computer-aided diagnosis (CADx) models utilizing CT images to predict malignant GGNs. These studies can be broadly categorized into two approaches. The first approach is based on radiomics features (6, 12, 13), which consists of a series of processes including tumor segmentation, radiomics feature extraction and selection, machine learning classifier training/testing, and performance evaluation (14–16). By utilizing radiomics models, thousands of quantitative imaging features are computed to decode the imaging phenotype of lung tumors. Although radiomics models can achieve high performance on limited datasets, tumor segmentation, and feature extraction are manually performed, which obviously cannot meet the requirements of clinical diagnosis. The other approach is the emergence of deep learning methods in recent years (17–20). Unlike radiomics models, deep learning-based models can extract CT image features using end-to-end deep neural networks and have achieved higher performance on large datasets (21–23). However, existing publicly available lung image datasets lack benign and malignant results confirmed by histopathology. Thus, these deep learning models can only predict the malignant tumor risk of GGNs rather than classify benign and malignant GGNs.

Reviewing relevant studies, we note that radiomics features can effectively decode the internal features of lung tumors, while deep learning-based imaging features can represent some features around the tumor (24). However, whether it is radiomics feature analysis methods or deep learning algorithms, they only fully utilize single-mode radiological data and ignore other modalities in cancer data, such as histopathology, genomics, or clinical information, making multimodal data integration relatively undeveloped (25, 26). In our previous research, we fused clinical, morphological, radiomics, and CT image features through BP neural networks (19), achieving higher performance compared to single-feature models, and preliminarily demonstrating the feasibility of multimodal features in the classification of benign and malignant GGNs.

To further optimize and improve the classification performance of the model, we introduced an attention mechanism to optimize the fusion mode of the four features. The attention mechanism is an effort to mimic the behavior of the human brain, selectively focusing on some important elements while ignoring others. Combining the attention mechanism with deep learning models helps automatically (through learning) focus on the most important parts of the input data. Therefore, theoretically, the attention mechanism can characterize the importance of different features through weight allocation, increasing the interpretability of the model. In this study, we explored the feasibility of using attention mechanisms to fuse clinical, morphological, radiomics, and CT image features to distinguish between benign and malignant GGNs.

## 2 Materials and methods

### 2.1 Datasets

The cohort was the same as the previous study (19). All GGNs were retrospectively collected from three medical institutions, respectively covering the periods from January 2019 to December 2021 (Hospital 1, Affiliated Hospital of Shandong Second Medical University), January 2016 to December 2018 (Hospital 2, Second Affiliated Hospital of Naval Medical University), and January 2020 to June 2022 (Hospital 3, The Second People’s Hospital of Deyang), and all were confirmed by pathology after thoracoscopy or open-chest surgery. Inclusion criteria were: (1) baseline GGNs (maximum cross-sectional diameter) > 5mm and ≤ 30mm; (2) baseline thin-section non-enhanced CT scan covering the entire lungs (slice thickness ≤ 2mm); and (3) CT scan performed within 1 month before surgery. Exclusion criteria were: (1) any form of anti-cancer treatment before surgery; (2) incomplete clinical or imaging data; (3) factors interfering with the display of GGNs, such as pseudo lesions; It should be noted that according to the 2021 classification recommendations of the World Health Organization, glandular precursor lesions (AAH, AIS) were classified as benign, while MIA and IAC were classified as malignant (27).

**Figure 1:**
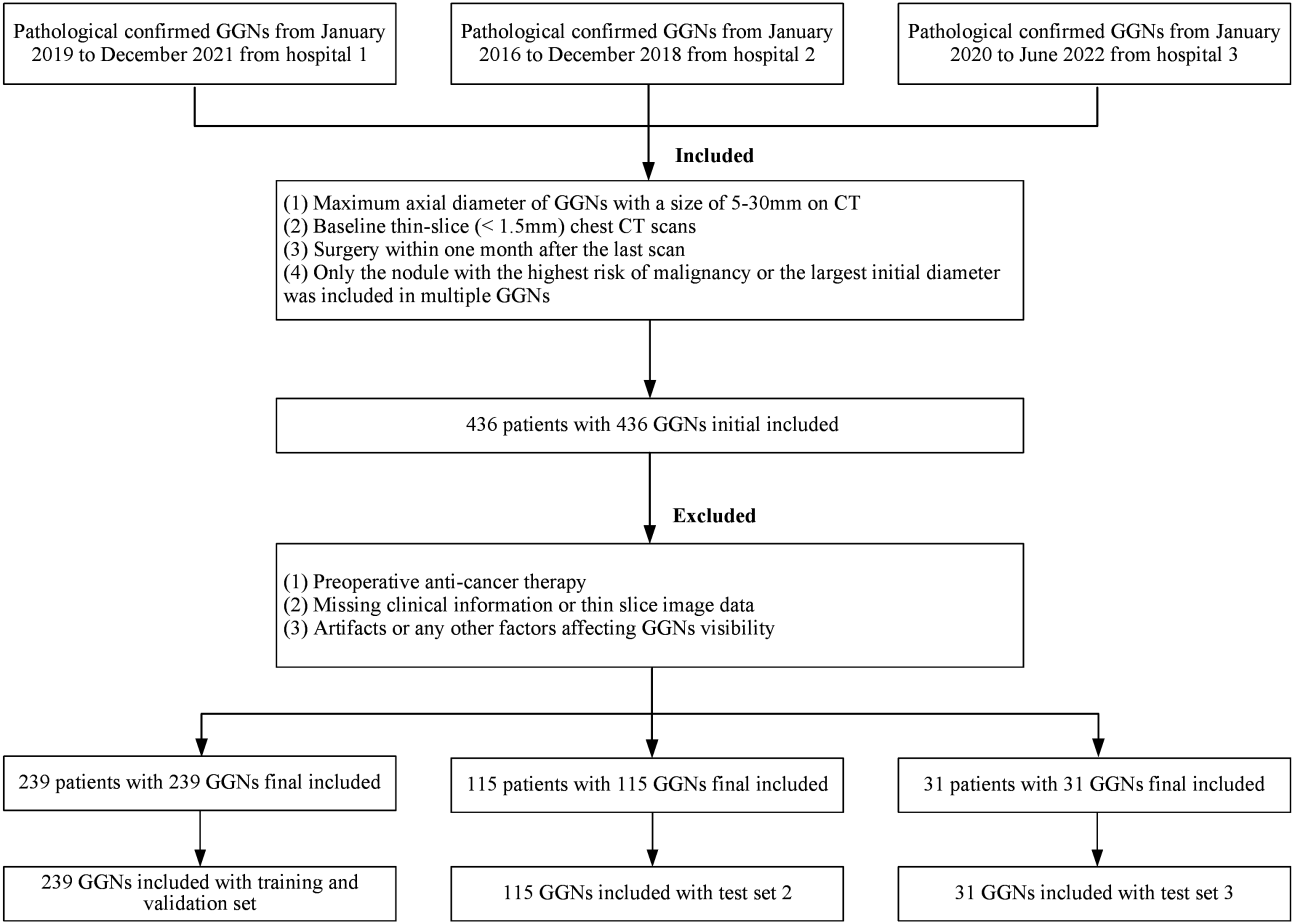
The inclusion and allocation of patients with pathological results.

In the end, a total of 385 GGNs from 385 patients (149 benign and 236 malignant) were included in the study (**Figure 1**). For the sake of training effectiveness and model generalization, we divided the data from the largest institution, Hospital 1 (239 patients, 239 GGNs, 60 Benign, 179 Malignant), into training and internal testing sets in a 6:4 ratio according to the proportion of benign and malignant cases, while the data from the smaller Hospital 2 (115 patients, 115 GGNs, 73 Benign, 42 Malignant) and Hospital 3 (31 patients, 31 GGNs, 16 Benign, 15 Malignant) were used as two independent external testing sets. Because of the retrospective nature of the study, the institutional review boards of the Hospital 2 approved the study without obtaining informed consent.

### 2.2 CT image

The CT scans were acquired by using multi-slice scanners with manufacturers of Siemens, Philips and GE medical systems. All CT images were retrieved from the picture archiving and communication system (PACS) and saved in digital imaging and communications in medicine (DICOM) format.

### 2.3 Clinical-morphological features evaluation

In this study, we meticulously gathered clinical information for all patients from the electronic medical record system. This encompassed four key clinical parameters: sex, age, smoking status, and family history of lung cancer. The evaluation of CT morphological features was conducted using specific settings for mediastinal (window width: 400 Hounsfield units [Hu], window level: -40 HU) and lung windows (1400 Hu, -600 HU). This task was independently undertaken by two adept chest radiologists (WH and XXZ, with seven and ten years of chest CT diagnostic experience, respectively), and subsequently cross-verified by another seasoned radiologist (LF, boasting 20 years of experience in this domain). Discrepancies in evaluations were harmonized through collaborative consultations. Notably, all radiologists conducted their assessments blinded to the pathological outcomes, ensuring an unbiased approach.

The CT morphological features scrutinized included the location, size, attenuation, shape, and margin of the nodules, as well as the nodule-lung interface, internal characteristics, and adjacent structures. The nodules were classified into three location types — inner, middle, and outer thirds of the lung — based on established quantitative definitions of central lung cancer. Size assessment involved measuring the maximum and minimum diameters on the axial section. Attenuation was categorized into either pure ground-glass nodules (pGGNs) or mixed ground-glass nodules (mGGNs), with pGGNs defined as areas of hazy increased lung attenuation and mGGNs as nodules comprising both ground-glass and solid components. The shapes were distinguished as either irregular or round/oval. Margin features included lobulation, spiculation, and a distinctive spine-like process, which is characterized by at least one convex border differing from the lung parenchyma boundary. The interface between the nodule and lung was categorized into ill-defined, well-defined and smooth, or well-defined but coarse. Internal features covered a range of aspects such as bubble lucency, cavitation, air-containing spaces, calcification, bronchial cut-off, and distorted/dilated bronchus (28, 29). Adjacent structures analysis included pleural indentation and vascular convergence. Additionally, the status of the bronchial wall and the presence of emphysema in the entire lung were meticulously evaluated, adding depth to our comprehensive assessment.

### 2.4 Whole lung segmentation and radiomics features

In this study, bilateral lung segmentation was meticulously executed using a publicly available 3D deep learning mode (30), effectively distinguishing lung tissue from the chest wall and mediastinum. This process was complemented by a manual revision to ensure precision in segmentation when necessary. Radiomics features were diligently extracted from the left, right, and bilateral lung tissues separately utilizing the Pyradiomics library (version 3.0) (14).

To uphold the highest standards of reproducibility and reliability, all radiomics feature extraction was conducted in strict adherence to the guidelines set forth by the Image Biomarker Standardization Initiative (IBSI) (31). To mitigate any variances that could arise from different scanner acquisitions, a thorough preprocessing of the acquired images was undertaken. This included normalization, resampling to a uniform voxel size of 1×1×1 mm³ using B-Spline interpolation, and gray-level discretization with a fixed bin width of 25.

From the original CT images, a comprehensive set of 107 features were extracted, encompassing 14 shape-based, 18 first-order statistics, 24 gray-level cooccurrence matrix, 14 gray-level dependence matrix, 16 gray-level run-length matrix, 16 gray-level size zone matrix, and 5 neighboring gray-tone difference matrix features. Moreover, 14 image filters were judiciously applied to the original images, generating derived images from which additional features were extracted. In total, an impressive array of 1409 radiomics features were meticulously extracted, contributing to the depth and breadth of this cutting-edge radiomics study.

### 2.5 Architecture of the proposed model

**Figure 2:**
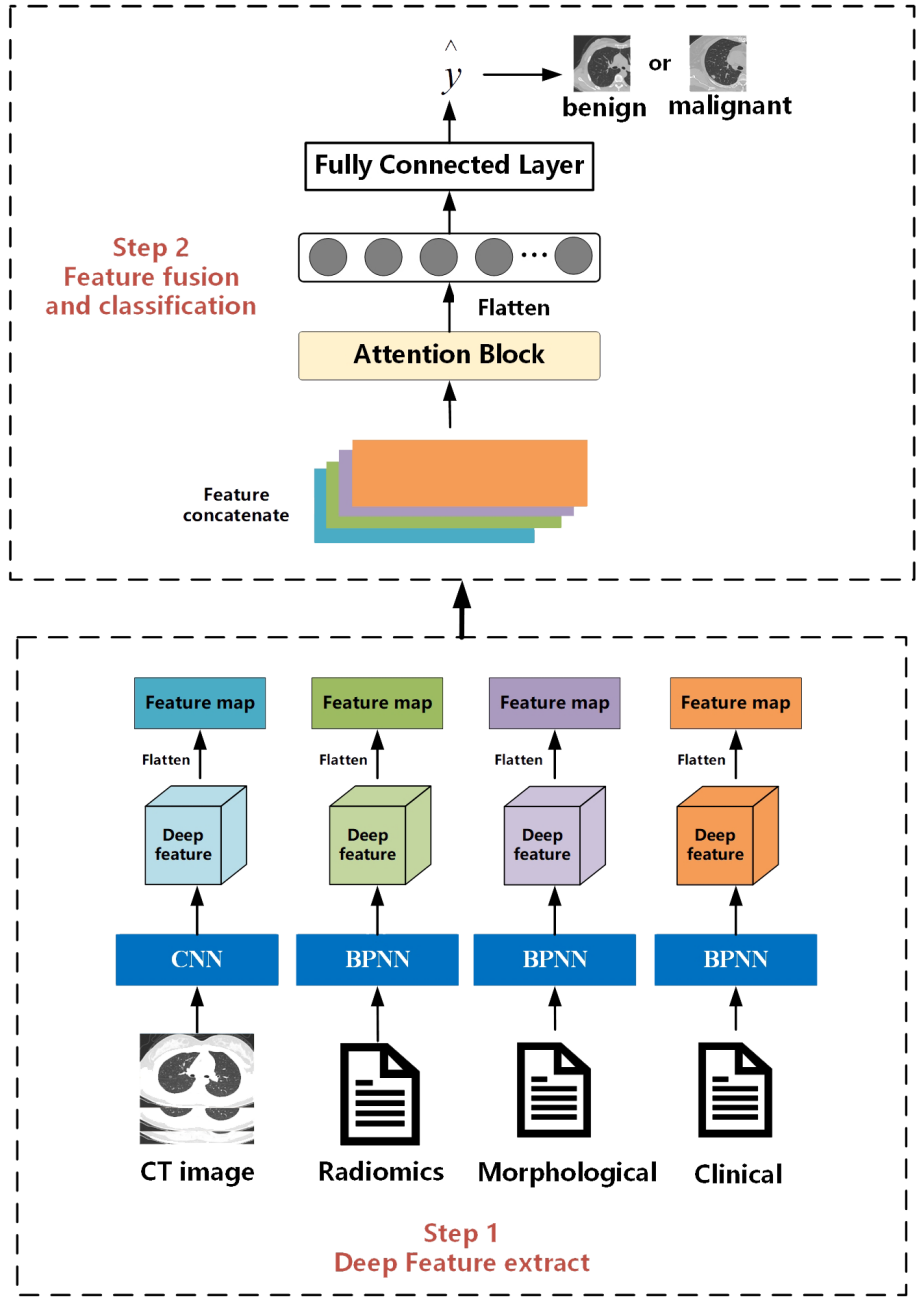
Flowchart of the proposed prediction model.

The overall workflow of this study is illustrated in **Figure 2**. The entire process consists of two steps. The first step is the feature extraction stage, which is the same as the work we have done in our previous research. Deep features are extracted separately from CT images, radiomics features, clinical, and morphological features using convolutional neural networks and BP neural networks. The second step is feature fusion and classification. Different from the previous work where only BP neural networks were used as the feature fusion algorithm, in this study, we innovatively introduce an attention mechanism. We hope that the model can learn the weight information between different features.

#### 2.5.1 Multi-feature extraction

For CT images, we designed a CNN with 26 layers to extract features, including 11 convolutional layers, 11 pooling layers, and 4 fully connected layers. Before inputting into the network, we first used a seed point algorithm to fill the lungs, obtaining the internal structure of the lungs, and then extracting the entire lung image and its internal tissue features. According to the description of the location of nodules in morphological features, the corresponding lung specimens were selected. Subsequently, the samples were formatted into full-lung images of 256*256*256 pixels. Finally, these images were input into the designed CNN, which outputs a 4*4*4 feature matrix.

For clinical data, morphological features, and whole-lung radiomics features, we designed networks with different numbers of layers based on the complexity of the data. For morphological and radiomics features with more variables, we used a BP neural network with 25 layers. For clinical features with fewer variables, we used a BP neural network with 5 layers. The BP neural network consists of a convolutional block and a fully connected layer. Before inputting into the network, we cleaned and processed text items (clinical data, morphological features) and whole-lung radiomics features. To facilitate input into the network, all text items were replaced by numbers. Then, z-score standardization was applied to process the whole-lung radiomics features with huge data dispersion to prevent challenges in obtaining features or fitting due to large dispersion when entering the network. Through the BP network, matrices of size 1*64 were outputted uniformly.

#### 2.5.2 Feature fusion and classification

The feature fusion and classification part consists of an attention module and an MLP layer. Extracted multimodal features from images, radiomics, clinical data, etc., are flattened into vectors of length 64 and concatenated into an 64*4 deep feature matrix. The attention module computes the weight proportions of different features. Subsequently, the deep feature matrix is fed into fully connected and softmax layers to output a value between [0, 1], indicating the probability of nodules being malignant. Then, by comparing this value with the threshold obtained during training, nodules are classified as benign or malignant. Nodules with values higher than the threshold are classified as malignant, while those with values lower than the threshold are classified as benign.

**Figure 3:**
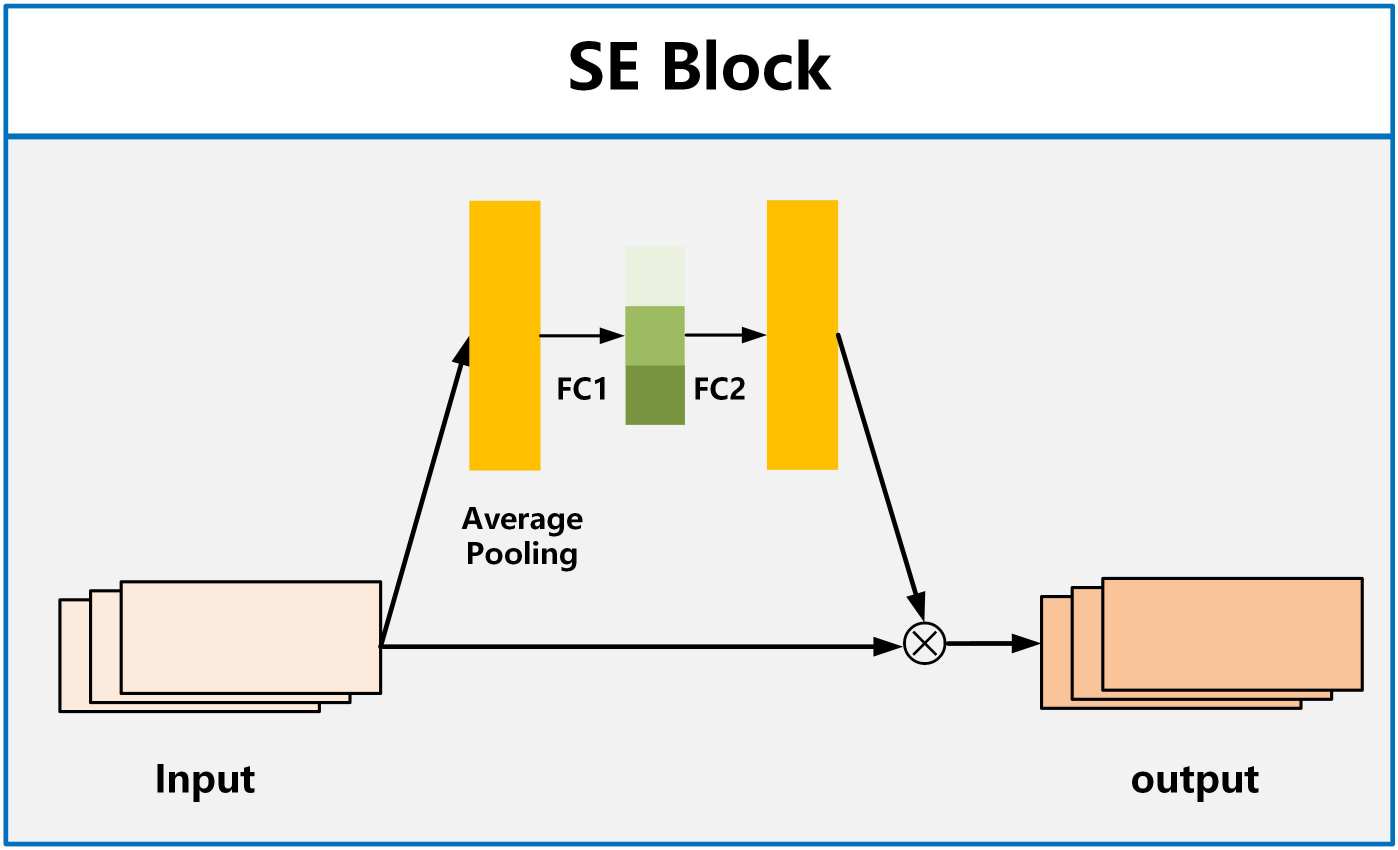
The structure of SE Block.

For the attention module, we employ the SE Block (Squeeze and Excitation Blocks) proposed by Hu et al. (32). SE Block has shown excellent performance in image classification, aiming to enhance the channel attention of the model (33, 34). It recalibrates features by strengthening useful features and weakening irrelevant features, driving the network to learn feature weights based on loss, thereby increasing the weights of effective feature maps and reducing the weights of ineffective or less effective feature maps. The SE Block consists of two fully connected layers named Squeeze and Excitation, as shown in **Figure 3**. The function of the Squeeze layer is to compress the input multi-dimensional feature layer and integrate the information of all feature layers. The function of excitation is to obtain the information dependence of each feature layer.

## 3 Result

### 3.1 Implementation details

#### 3.1.1 Experimental settings

For classification, we used the weighted sum of binary cross-entropy loss as loss function. Cross-entropy has been widely used in general classification tasks due to its robustness. We used stochastic gradient decent (SGD) as optimizer, with an initial learning rate of 0.001, which was reduced when the metric did not improve with patience of 15. The mini-batch size was set to 32. The Max epoch number was set to 1000 and early stopping with a patience of 200 was used.

#### 3.1.2 Model evaluation

To evaluate the model’s predictive capability, the probability of each output was compared with the corresponding label. The prediction performance was evaluated using the area under the receiver operating characteristic curve (AUC), sensitivity (SEN), and specificity (SPE). The positive predictive value (PPV), the negative predictive value (NPV), and accuracy (ACC) were also used to assess model performance. P values <0.05 were considered significant.

All performance evaluation processes were performed in Python (version 3.6.8, Python Software Foundation, USA) environment by using a computer configured with Intel Core i9-13900K CPU, 64 GB RAM, and NVIDIA GeForce RTX 4090 graphics processing unit.

### 3.2 Experimental results

#### 3.2.1 Results of feature fusion for four features across three datasets

**Figure 4:**
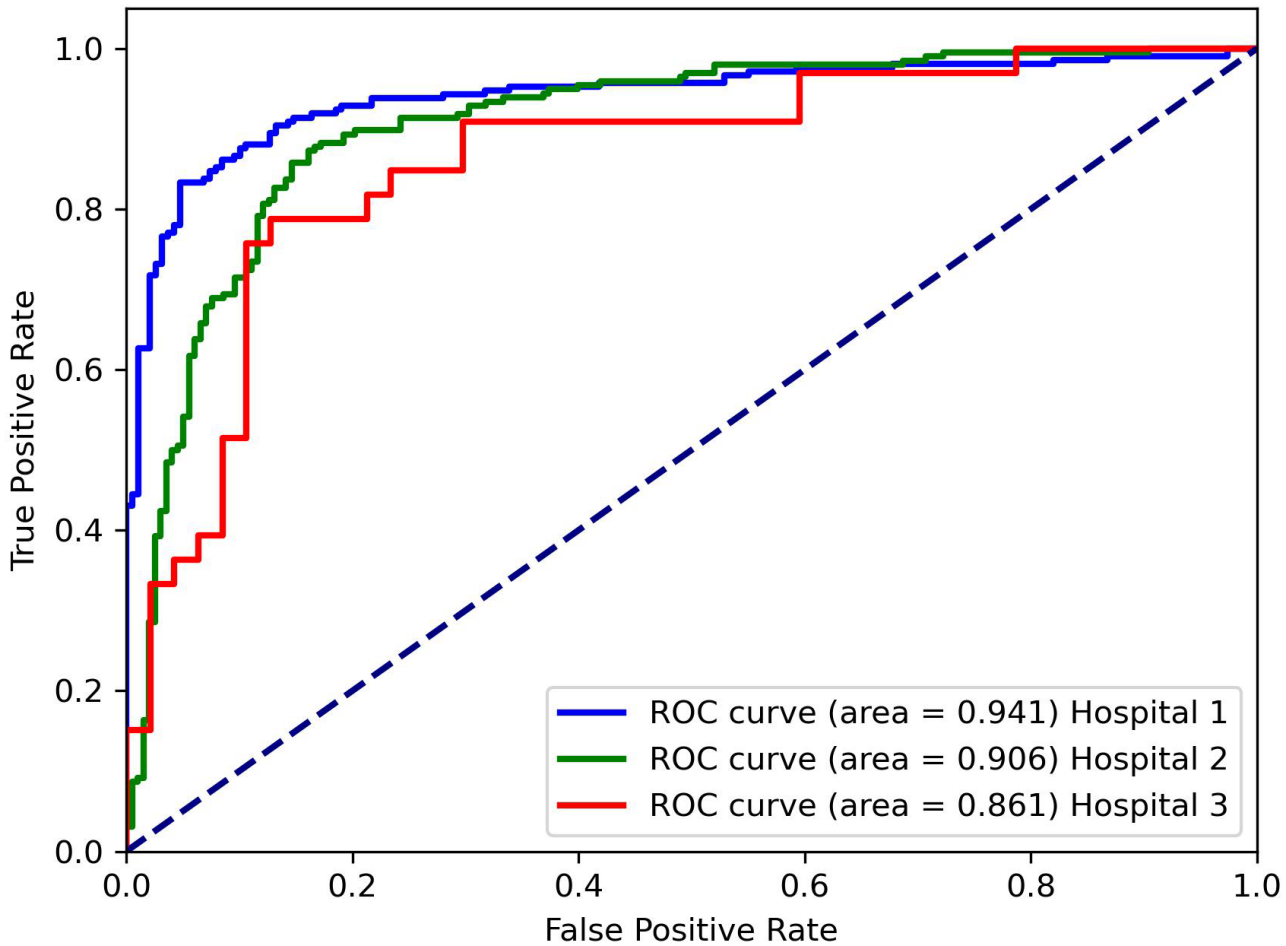
Receiver operating characteristics (ROC) curve for three hospital datasets.

**Figure 5:**
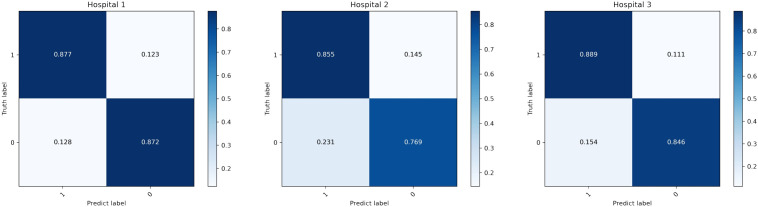
Corresponding confusion matrix for three hospital datasets.

**Table 1** presents the classification results of the models on different datasets. For the testing dataset from Hospital 1, the model achieved an accuracy of 0.875, sensitivity of 0.872, specificity of 0.877, and AUC of 0.941. For the testing dataset from Hospital 2, the model achieved an accuracy of 0.826, sensitivity of 0.769, specificity of 0.855, and AUC of 0.861. For the testing dataset from Hospital 3, the model achieved an accuracy of 0.871, sensitivity of 0.846, specificity of 0.889, and AUC of 0.906. The ROC curves for the three datasets are shown in **Figure 4**, and the corresponding confusion matrices are shown in **Figure 5**. The ROC curves indicate that the AUC for the dataset from Hospital 2 is lower than that of Hospital 1 and Hospital 3. Additionally, there are differences in model performance among different datasets, specifically, Hospital 2 performs better than Hospital 3.

**Table 1:** Classification performance of the CMRI-AM model in three hospital datasets with pathologically confirmed GGNs.

Data	AUC	ACC	SEN	SPC	PPV	NPV
Hospital 1	0.941	0.875	0.872	0.877	0.829	0.909
Hospital 2	0.861	0.826	0.769	0.855	0.731	0.878
Hospital 3	0.906	0.871	0.846	0.889	0.846	0.842

#### 3.2.2 Classification results of different feature fusion methods

**Table 2** lists the AUC values and corresponding 95% confidence intervals (CI) generated by different feature fusion methods. By comparing the performance of different feature combination models, the fusion of clinical, morphological, radiomics, and image features achieved the highest performance. The model produced an AUC value of 0.912 (95% CI: [0.848, 0.972]), which was higher than all other feature combination methods. To further analyze the performance of the fusion model, we also calculated the AUC difference between the 4 models that fused 3 different features (C-M-I, C-M-R, M-R-I, C-R-I) and the model that fused all features(C-M-R-I). **Table 3** lists the details.

**Table 2:** AUC values and corresponding 95% CIs generated by different feature combinations in three hospital datasets.

Feature combination	Hospital 1		Hospital 2		Hospital 3	
	AUC	95% CI	AUC	95% CI	AUC	95% CI
C-M	0.836	[0.797, 0.865]	0.806	[0.717, 0.763]	0.809	[0.767, 0.823]
M-R	0.848	[0.815, 0.889]	0.798	[0.735, 0.787]	0.818	[0.789, 0.837]
M-I	0.842	[0.786, 0.872]	0.826	[0.768, 0.819]	0.846	[0.798, 0.869]
C-R	0.835	[0.813, 0.883]	0.808	[0.779, 0.823]	0.838	[0.809, 0.813]
C-I	0.827	[0.785, 0.864]	0.827	[0.772, 0844]	0.849	[0.811, 0.874]
R-I	0.832	[0.771, 0.865]	0.813	[0.781, 0.851]	0.845	[0.821, 0.911]
C-M-I	0.873	[0.829, 0.903]	0.823	[0.785, 0.873]	0.873	[0.849, 0.903]
C-M-R	0.895	[0.832, 0.912]	0.815	[0.779, 0.868]	0.865	[0.832, 0.898]
M-R-I	0.925	[0.856, 0.939]	0.841	[0.846, 0.892]	0.879	[0.846, 0.902]
C-R-I	0.917	[0.858, 0.943]	0.833	[0.818, 0.873]	0.877	[0.823, 0.923]
C-M-R-I	0.941	[0.898, 0.972]	0.861	[0.823, 0.882]	0.906	[0.878, 0.932]

C, clinical; M, morphological; R, radiomics; I, image; AUC, area under the operator characteristic curve; 95% CI, 95% confidence interval.

**Table 3:** Comparison of AUC differences between different feature fusion models and the C-M-R-I model.

Feature combination	Hospital 1	Hospital 2	Hospital 3
C-M-I	0.007	0.032	0.041
C-M-R	0.018	0.019	0.028
M-R-I	0.047	**0.064**	0.039
C-R-I	0.035	0.042	0.037

Data with no significant difference are shown in bold.

## 4 Discussion

The widespread use of high-resolution CT has made it increasingly easier to detect GGNs. However, the slow growth and atypical morphological features of GGNs also make it more challenging to distinguish between benign and malignant GGNs (35, 36). Currently, most artificial intelligence (AI) models used to predict benign and malignant pulmonary nodules are constructed based on local features of nodules or a combination of features within a specific range around the nodules.

In this study, we proposed a deep-learning model that utilizes attention mechanisms to fuse multimodal whole-lung features for the classification of benign and malignant GGNs. This represents a further improvement from our previous work (19). To the best of our knowledge, in existing studies on distinguishing benign and malignant nodules, almost all focus on mining radiomics features from CT images or segmenting and analyzing original CT images, while ignoring histopathological, genomic, or clinical information in cancer data, resulting in inefficient utilization of multimodal data. In the work of feature fusion, Hu et al. proposed a computer-aided diagnosis of ground-glass lung nodules by fusing deep learning and radiomics features (24), Xia et al. also used same method (37). Wang et al. proposed a fusion diagnostic model integrating the original images and the clinical and image features (38). Compared to single CT feature, their multi-features fusion model achieved significant improvements in multiple indicators, demonstrating the great potential of multimodal features in predicting benign and malignant GGNs.

**Figure 6:**
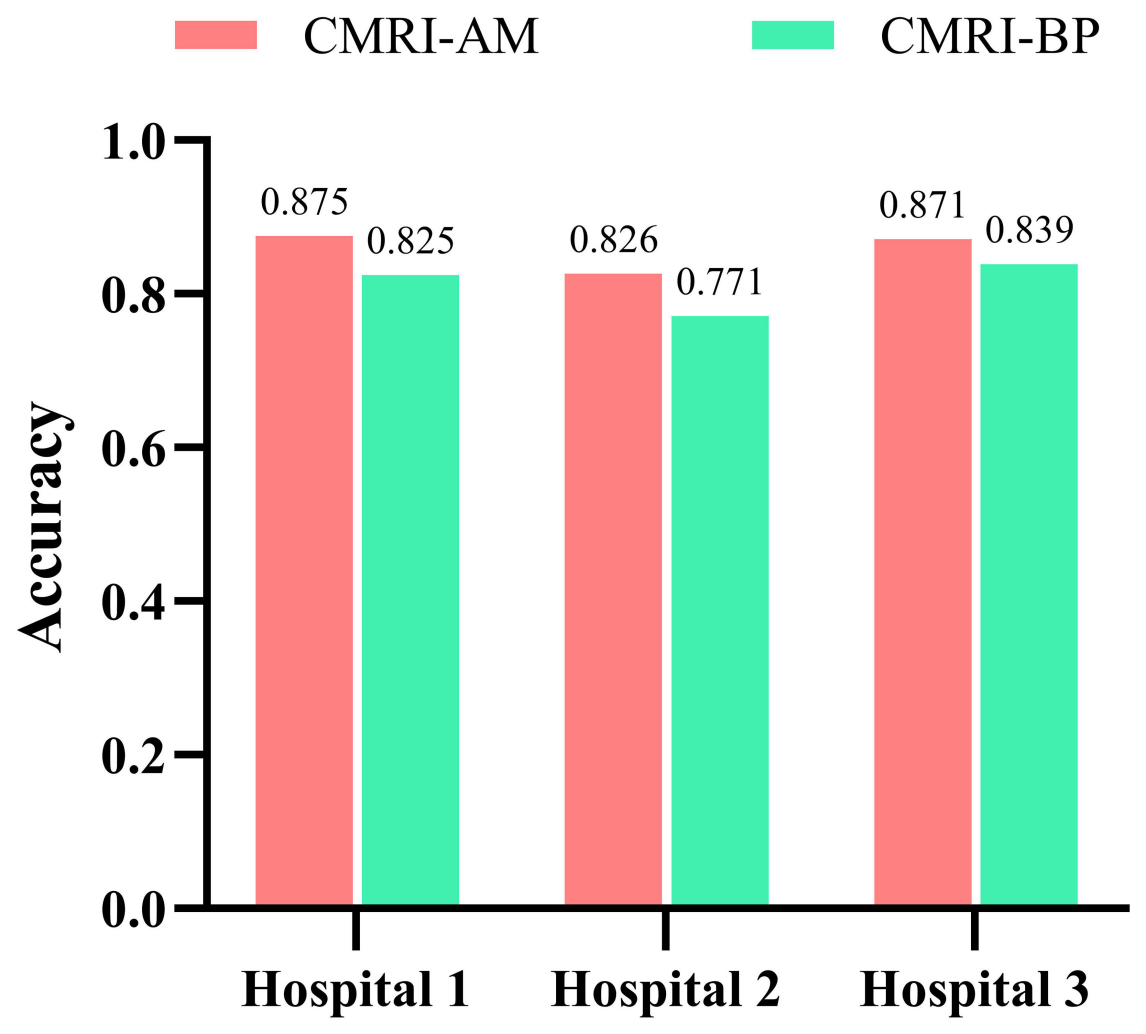
Comparison of the accuracy of two feature fusion methods on three data sets.

**Figure 7:**
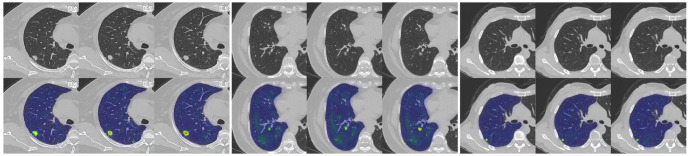
An example of model GRAD-CAM heat map. The top image represents the original CT image, and the bottom image represents the class activation map (CAM). In the CAM image, regions highlighted in red or yellow indicate high importance or strong activation, while regions in blue or green indicate low importance or weak activation.

Our study has several characteristics. Technically, we mainly improved in two aspects. Firstly, we introduced an attention mechanism based on the linear BP network to fully learn the weight relationships between the four features: clinical, morphological, image, and radiomics, enabling the model to selectively focus on important features. Secondly, to address the problem of model robustness and generalization due to the small sample size, we adopted data augmentation methods from previous studies, such as sample translation and rotation, to increase the diversity of samples (38, 39). In terms of specific details, before fusing multimodal features, we utilized deep CNNs to extract deep image features of CT to capture high-dimensional representations of various features. For clinical, morphological, and radiomics features, we designed different numbers of layers of feedback neural networks to expand them into high-dimensional spaces. This approach resolved the problem of difficulty in fusing multimodal features in high-dimensional feature space. Additionally, compared to linear neural networks, the attention mechanism showed better feature fusion effects, as shown in **Figure 6**, where classification accuracy increased by 3.5%, 2.7%, and 4.6% on datasets from three different hospitals. Regarding model interpretability, we utilized class activation maps to visualize the features learned by the model. Class activation map (CAM) is a technique to visualize the regions of input data that are important for predictions from CNN-based models (40). The results demonstrated that the model effectively learned local nodule features in whole-lung images and discriminated between the benign and malignant nature of GGNs through complementary information from other features. (**Figure 7**).

Although we achieved good results, our study has some limitations. Firstly, our retrospective study was conducted using an imbalanced and limited dataset, All GGNs were confirmed by postoperative pathology. These nodules were biased towards a diagnosis of malignancy, resulting in fewer benign GGNs than malignant ones, making selection bias inevitable. As the DNN model is a data-driven algorithm, a small training dataset may lead to underfitting issues. Although many data augmentation techniques were applied to increase the number of training samples, the lack of training samples may reduce the model’s performance. The proposed model was only validated and tested on a limited private testing dataset. Therefore, it is necessary to verify and test the model’s performance using more diverse and larger multicenter datasets. Secondly, the features fused by our model are all based on whole-lung extraction, without comparison with models based on local nodule features. Further research is needed to determine whether our model has advantages over models based on local nodule features. Thirdly, there may be other smaller GGNs present in the same lung as the target GGN and the features of these small GGNs may affect the predictive performance of the model. Finally, this is only a technological development study. We were fortunate to demonstrate that the attention mechanism can effectively improve the fusion efficiency of multimodal features in whole-lung data. However, before applying this model to clinical practice, it should be validated in more clinical datasets.

## 5 Conclusion

In this work, we utilized an attention mechanism to integrate multimodal tumor features, including clinical information, morphological features, radiomics features, and whole-lung CT images, effectively improving the identification of benign and malignant nodules. Experimental results demonstrate that: (1) the application of the attention mechanism to integrate whole-lung CT images, radiomics features, clinical, and morphological features is feasible, (2) clinical, morphological, and radiomics features provide complementary information for the classification of benign and malignant GGNs based on CT images, and (3) utilizing baseline whole-lung CT features to predict the benign and malignant nature of GGNs is an effective approach. Therefore, by optimizing the fusion of baseline whole-lung CT features, the classification performance of GGNs’ benign and malignant nature can be effectively improved. Lastly, we hope this research will inspire further studies to enhance model performance collaboratively, therebypromoting the application of artificial intelligence in clinical diagnostics.

## Data Availability

The data analyzed in this study is subject to the following licenses/restrictions: The original contributions presented in the study are included in the article. Further inquiries can be directed to the corresponding author. Requests to access these datasets should be directed to fanli0930@163.com.
